# Highly Pathogenic Avian Influenza Virus (H5N1) Clade 2.3.4.4b Introduced by Wild Birds, China, 2021

**DOI:** 10.3201/eid2907.221149

**Published:** 2023-07

**Authors:** Jingman Tian, Xiaoli Bai, Minghui Li, Xianying Zeng, Jia Xu, Peng Li, Miao Wang, Xingdong Song, Zhiguo Zhao, Guobin Tian, Liling Liu, Yuntao Guan, Yanbing Li, Hualan Chen

**Affiliations:** State Key Laboratory for Animal Didease Control and Prevention, Harbin Veterinary Research Institute, Chinese Academy of Agricultural Sciences, Harbin, China (J. Tian, X. Bai, M. Li, X. Zeng, X. Song, Z. Zhao, G. Tian, L. Liu, Y. Guan, Y Li, H. Chen);; Preventive and Control Center for Animal Disease of Heilongjiang Province, Harbin (J. Xu, P. Li, M. Wang)

**Keywords:** influenza, influenza virus, clade 2.3.4.4b, H5N1, wild birds, phylogeny, virulence, antigenic property, protective efficacy, viruses, China

## Abstract

Highly pathogenic avian influenza (HPAI) subtype H5N1 clade 2.3.4.4b virus has spread globally, causing unprecedented large-scale avian influenza outbreaks since 2020. In 2021, we isolated 17 highly pathogenic avian influenza H5N1 viruses from wild birds in China. To determine virus origin, we genetically analyzed 1,529 clade 2.3.4.4b H5N1 viruses reported globally since October 2020 and found that they formed 35 genotypes. The 17 viruses belonged to genotypes G07, which originated from eastern Asia, and G10, which originated from Russia. The viruses were moderately pathogenic in mice but were highly lethal in ducks. The viruses were in the same antigenic cluster as the current vaccine strain (H5-Re14) used in China. In chickens, the H5/H7 trivalent vaccine provided complete protection against clade 2.3.4.4b H5N1 virus challenge. Our data indicate that vaccination is an effective strategy for preventing and controlling the globally prevalent clade 2.3.4.4b H5N1 virus.

The hemagglutinin (HA) gene of highly pathogenic avian influenza subtype H5 viruses has evolved into multiple clades (clades 0–9), and some clades are further divided into subclades. Of the 2 dominant clades, 2.3.2.1 has been further categorized into 7 (2.3.2.1a–g) and 2.3.4.4 have been further categorized into 8 (2.3.4.4a–h) subclades (*1*–*3*). The World Organisation for Animal Health reported that >8,000 outbreaks of highly pathogenic avian influenza (HPAI) subtype H5N1 clade 2.3.4.4b occurred in birds during October 2020–October 2022. Massive numbers of birds across 4 continents (Europe, Asia, Africa, and North America) were humanely killed directly or indirectly by infection with clade 2.3.4.4b H5 HPAI viruses (*4*).

H5 HPAI viruses of several clades have spread intercontinentally through global migration of wild birds. In 2005, clade 2.2 H5N1 virus spread by wild birds caused numerous outbreaks in wild birds and domestic poultry in countries throughout Asia, the Middle East, Europe, and West Africa (*5*,*6*). In 2009, clade 2.3.2 H5N1 virus caused problems mainly in Asia and eastern Europe (*7*,*8*). In 2014, both clade 2.3.4.4b H5N8 and clade 2.3.2.1c H5N1 viruses spread and circulated in Eurasia, the Middle East, and Africa (*9*–*11*). At the beginning of 2014, a clade 2.3.4.4c H5N8 virus emerged in South Korea and then circulated in Eurasia and Africa. In 2015, that same virus spread to North America and reassorted with local low pathogenicity avian influenza (LPAI) viruses to produce subtype H5N2, which circulated in the United States during 2015–2016 (*12*,*13*). At the beginning of 2020, clade 2.3.4.4b H5N8 virus caused disease outbreaks and destroyed poultry across Europe, after which it spread to many countries in Asia (*14*,*15*). The H5N8 virus reassorted with different viruses and formed several other subtypes of H5 viruses (e.g., H5N1, H5N2, H5N3, H5N4, H5N5, and H5N6) in different countries and regions. Among them, H5N1 became the globally predominant variant (*16*,*17*). In late 2021, the virus was carried across the Atlantic Ocean to North America (*18*). The viruses caused huge ongoing outbreaks in Europe and North America and led to massive destruction of poultry and wild bird populations (*4*,*19*,*20*).

Clade 2.3.4.4b H5 viruses have crossed the bird–mammal barrier to infect humans and other mammals. Since the beginning of 2020, human infection with influenza A(H5N1) clade 2.3.4.4b viruses has been detected in Spain, the United Kingdom of Great Britain and Northern Ireland, the United States, China, and Vietnam and reported to the World Health Organization (*21*). Seven human infections caused by influenza A(H5N8) virus were reported in the Russian Federation (*22*), and some infections caused by H5N6 virus were reported in China in those 3 years (2020–2022) (*23*). In addition, fatal 2.3.4.4b H5N1 infection of some carnivorous mammals (e.g., foxes, otters, red foxes, skunks, coyotes, bobcats) and marine mammals (e.g., harbor seals, dolphins) has been reported in Europe and North America (*24*).

Clade 2.3.4.4b H5N1 virus has become a new threat to the global poultry industry and to public health. To learn more about its spatial transmission and biological properties, we performed extensive phylogeographic and epidemiologic analyses of the globally circulating H5N1 viruses detected during 2020–2022, evaluated the pathogenicity of H5N1 from China in mammalian and waterfowl hosts, compared H5N1 antigenicity with that of the updated vaccine candidate, and assessed the protective efficacy of the current H5-Re14 vaccine against challenge with H5N1 isolates. 

All studies with live viruses were conducted in a Biosafety Level 3 laboratory approved for such use by the Harbin Veterinary Research Institute of the Chinese Academy of Agricultural Sciences. All experiments using animals were conducted in strict accordance with recommendations in the Guide for the Care and Use of Laboratory Animals of the Ministry of Science and Technology of the People’s Republic of China. The protocol was approved by the Committee on the Ethics of Animal Experiments of the Harbin Veterinary Research Institute of the Chinese Academy of Agricultural Sciences (approval nos.: duck, 211015-01; mouse, 211231-02; chicken, 211112-01).

## Materials and Methods

### Sample Collection and Virus Isolation

During 2020–2021, we collected 7,421 fresh fecal samples, 507 swab samples, and 6 tissue samples from wild birds in accordance with the regular surveillance of wild birds in China (Table). The samples were amplified in specific-pathogen–free (SPF) chicken embryos, and the HA subtype was identified by using the hemagglutinin inhibition (HI) test with a panel of H1–H16 reference serum. We verified the neuraminidase (NA) subtype by using reverse transcription PCR analysis with a panel of N1–N9 subtype primers (reference serum and primer sequences available on request). We identified host species by using DNA barcoding with the cytochrome C oxidase I mitochondrial gene (*25*).

**Table:** Avian influenza viruses isolated from wild bird samples collected in China, January 2020–December 2021*

Date	Province	Sample				Virus		
		Feces	Swab	Tissue		No. strains	Subtype	Pathotype†
2020								
Jan	Shanxi	700	0	0		2	H5N3	Low
Jan	Heilongjiang	980	394	2		0	NA	NA
Nov	Anhui	1,017	0	0		17	Multiple‡	Low
Nov	Ningxia	600	0	4		1	H5N2	Low
2021								
Mar	Anhui	700	0	0		0	NA	NA
Mar	Liaoning	600	0	0		0	NA	NA
Apr	Ningxia	450	0	0		0	NA	NA
Apr	Heilongjiang	750	0	0		0	NA	NA
Oct	Heilongjiang	324	113	0		2	H5N1	High
Dec	Shanxi	500	0	0		4	H5N1	High
Dec	Henan	800	0	0		11	H5N1	High

*NA, not applicable. †Pathotype is determined by the cleavage motif of its hemagglutinin gene. ‡The 17 low pathogenicity viruses are 1 H2N3, 1 H3N8, 9 H4N6, 3 H5N8, 1 H6N2, 1 H6N8, and 1 H12N2 strains.

### Genome Sequencing and Phylogenic and Phylodynamic Analyses

We extracted total influenza A virus RNA from the allantoic fluid of virus-infected chicken embryos by using the QIAmp Viral RNA Mini Kit (QIAGEN, https://www.qiagen.com). We performed reverse transcription PCR by using a panel of gene-specific primers and sequenced the products by using an Applied Biosystems DNA analyzer (primers available on request). The genetic information collected from January 1, 2020, to October 17, 2022, was downloaded from GISAID (https://www.gisaid.org) on October 17, 2022 (Appendix 1). We aligned sequences by using MAFFT version 7.475 with default settings (*26*) and ran neighbor-joining trees by using MEGA version 11 for 1,000 ultrafast bootstraps (*27*). To categorize the groups of each segment in the phylogenetic trees, we used a sequence identity cutoff of >95%. We constructed a maximum-clade credibility time-scaled phylogenetic tree of HA sequences from clade 2.3.4.4b H5N1 viruses by using the SRD06 nucleotide substitution model and an uncorrelated lognormal relaxed clock model (*28*). To investigate the transmission patterns of the clade 2.3.4.4b H5N1 viruses, we performed a phylogeographic analysis by using an asymmetric model with Bayesian stochastic search variable selection implemented in BEAST version 1.10.4 (*28*,*29*). We grouped the sequences from 1,529 isolates into 11 distinct geographic categories (Appendix 2 Table 3). To summarize the diffusion rates, we used the Bayesian stochastic search variable selection, and to estimate Bayes factors, we used SpreaD3 version 0.9.6 (https://rega.kuleuven.be/cev/ecv/software/SpreaD3) (Appendix 2 Table 4).

### Animal Studies

We selected 2 representative isolates, A/mandarin duck/Heilongjiang/HL-1/2021 (MD/HLJ/HL-1/2021) and A/whooper swan/Henan/14/2021 (WS/HeN/14/2021), to test in mice and ducks. The 50% lethal dose (LD_50_) for mice was tested in groups of five 6-week-old female BALB/c mice (Vital River, https://www.vitalriver.com). The mice were intranasally inoculated with a 10^1.0^ to 10^6.0^ 50% egg infectious dose (EID_50_) of virus in a volume of 50 µL and then monitored daily for weight loss and death for 14 days. LD_50_ values for mice were calculated according to the Reed-Muench method (*30*). To evaluate virus replication, we tested 3 more mice in the 10^6.0^ EID_50_ group and humanely killed them on postinoculation day 3 to assess virus titers in their nasal turbinates, lungs, brains, kidneys, and spleens.

Among the ducks, we inoculated groups of eight 3-week-old SPF ducks (Jinding duck, a local breed; National Poultry Laboratory Animal Resource Center, Harbin, China) intranasally with 10^6.0^ EID_50_ of H5N1 virus in a volume of 0.1 mL; at 24 h after inoculation, 3 contact ducks were put in the same cage. On postinoculation day 3, we randomly selected 3 of 8 infected ducks, humanely killed them, and collected their organs (brain, spleen, kidneys, pancreas, cecal tonsil, bursa of Fabricius, thymus, lungs, and larynx) for virus titration. We observed the remaining 5 infected ducks and 3 contact ducks for 2 weeks and collected oropharyngeal and cloacal swab samples on postinoculation days 3 and 5 to detect virus shedding.

### Antigenic Analysis

We used HI to perform antigenic analysis (*31*). We generated chicken antiserum of H5 vaccine seed viruses (H5-Re11, H5-Re12, H5-Re13, and H5-Re14) (Appendix 2 Table 7) by inoculating 5-week-old SPF chickens with 0.3 mL of the oil-emulsified inactivated viruses (*32*).

### Challenge Study of Clade 2.3.4.4b H5N1 Virus in Chickens

We vaccinated groups of ten 3-week-old white leghorn SPF chickens (National Poultry Laboratory Animal Resource Center) with a 0.3-mL intramuscular injection of the trivalent H5/H7 vaccine previously reported by Zeng et al. (*32*,*33*) (Harbin Weike Biotechnology Co., http://www.hvriwk.com) or with phosphate-buffered saline as a control. Three weeks after vaccination, we challenged the chickens intranasally with 10^5^ EID_50_ of MD/HLJ/HL-1/2021 in a volume of 0.1 mL. We collected oropharyngeal and cloacal swab samples on postchallenge days 3 and 5 to detect the virus and observed birds for disease and death for 2 weeks after challenge.

## Results

### Clade 2.3.4.4b H5N1 Virus Detection 

As part of regular surveillance of wild birds during 2020–2021, we collected 7,934 samples, from which we detected 17 H5N1 HPAI viruses and 20 LPAI viruses of multiple subtypes (Table). To determine the evolution of the H5N1 viruses, we sequenced the whole genomes of the 17 HPAI viruses, deposited the sequences in the GISAID database (accession nos. EPI2070071–0206), and performed phylogenic analysis of these viruses together with global clade 2.3.4.4b HPAI H5 viruses submitted to GISAID from January 1, 2020, to October 17, 2022.

**Figure 1:**
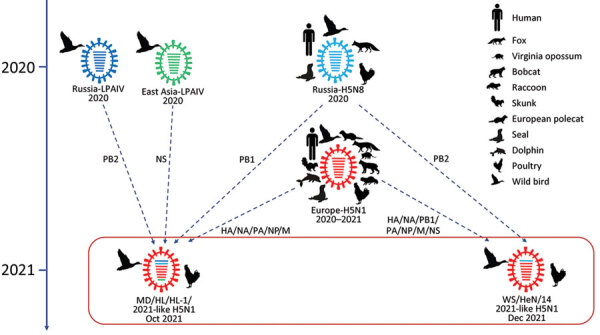
Formation of 2 representatives of avian influenza (H5N1) virus from China. Viral particles are represented by colored ovals containing horizontal bars representing the 8 gene segments (from top to bottom: PB2, PB1, PA, HA, NP, NA, M, and NS). Gene segments of the reassortants are colored according to their corresponding source virus. HA, hemagglutinin; LPAIV, low pathogenicity avian influenza virus; M, matrix; NA, neuraminidase; NP, nucleoprotein; NS, nonstructural protein; PA, polymerase acidic; PB, polymerase basic.

**Figure 2:**
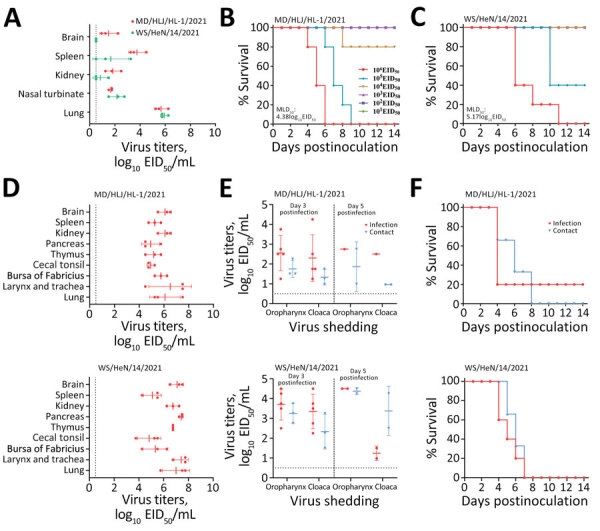
Replication and virulence of representative highly pathogenic avian influenza virus (H5N1) viruses in mice and ducks. A) Virus titers in organs of mice that were humanely killed on postinfection day 3 with 10^6^ EID_50_ of the test viruses. B, C) MLD_50_ of the indicated viruses. D) Virus titers in organs of ducks that were killed on day 3 post inoculation with 10^6^ EID_50_ in 0.1 mL of the indicated viruses. E) Virus shedding from ducks on the indicated days after inoculation. F) Death pattern of ducks in the infected and control groups. EID_50_, 50% egg infectious dose; MLD_50_, 50% lethal dose for mice.

**Figure 3:**
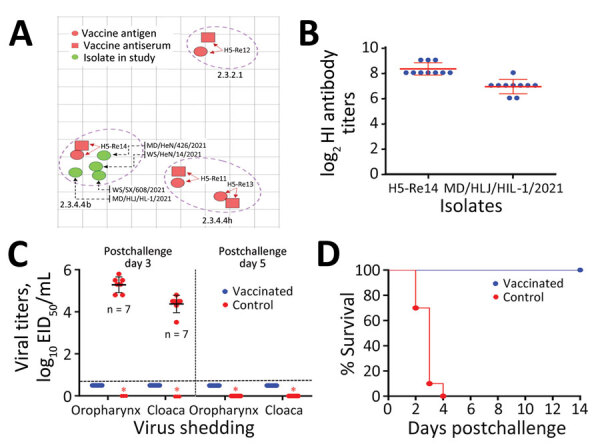
Antigenic difference of avian influenza (H5N1) virus isolates with H5 vaccine strains and protective efficacy of H5 vaccine against the challenge of H5N1 virus. A) Antigenic cartography of H5N1 viruses. The map was generated by using Antigenic Cartography software (https://acmacs-web.antigenic-cartography.org); 1 unit (grid) represents a 2-fold change in the HI assay results (Appendix 2 Table 7). B) HI antibody titers of vaccinated chickens against the vaccine seed virus and the challenge strain. C) Virus shedding from chickens on postchallenge days 3 and 5. D) Survival patterns of chickens in the vaccinated and control groups after challenge with the indicated H5N1 virus. EID_50_, 50% egg infectious dose; HI, hemagglutination inhibition.

The HA genes of the 17 H5N1 viruses shared 98.3%–100% identity at the nucleotide level, and the NA genes shared 97.45%–100% identity (Appendix 2 Figure 2, Figure 3, panel A). The HA genes of the clade 2.3.4.4b H5 strains formed 2 branches—East Asia and Eurasia/Africa—in the phylogenetic tree (Appendix 2 Figures 1, 2); 2 H5N1 viruses isolated in Heilongjiang Province in this study belonged to the East Asia branch, whereas the other 15 viruses belonged to the Eurasia/Africa branch. The NA genes of the 17 viruses all belonged to the group Europe H5N1 2020 (Appendix 2 Figure 3, panel A). Identity at the nucleotide level was 94.12%–100% for the polymerase basic (PB) 2 genes, 92.96%–100% for the PB1 genes, 97.3%–100% for the polymerase acidic (PA) genes, 97.6%–100% for the nucleoprotein (NP) genes, 98.7%–100% for the matrix (M) genes, and 95.65%–100% for the nonstructural protein (NS) genes of the 17 H5N1 viruses, and they formed multiple groups in their phylogenetic trees (Appendix 2 Figure 3). Diversity of the internal genes of clade 2.3.4.4b H5N1 viruses was greater than that of the HA and NA genes. Of note, the 8 genes of the MD/HLJ/HL-1/2021 and MD/HLJ/HL-2/2021 viruses were similar, and the 8 genes of the other 15 viruses were similar, indicating that these 17 viruses formed 2 different genotypes.

### Complex Reassortment of the H5N1 Wild Bird Viruses 

To investigate the formation of the 17 H5N1 HPAI viruses, we used MD/HLJ/HL-1/2021 and WS/HeN/14/2021 as representatives of the 2 branches and conducted BLAST analysis (https://platform.epicov.org/epi3/frontend#27b74d) of the highest homology viruses to the 8 segments of 2 reference viruses from GISAID. The data showed that both representatives were reassortants (Figure 1; Appendix 2 Table 2). Seven genes of WS/HeN/14/2021 (PB1, HA, NA, PA, NP, M, and NS) were closely related to those of the H5N1 HPAI viruses in Europe during 2020–2021; the PB2 gene of WS/HeN/14/2021 was provided by the H5N8 HPAI virus in Russia in 2020. MD/HLJ/HL-1/2021 had 5 genes (HA, NA, PA, NP, and M) closely related to those of the H5N1 HPAI viruses in Europe during 2020–2021; its PB1 gene was closely related to the H5N8 HPAI virus in Russia during 2020, whereas its PB2 and NS genes were from the LPAI viruses that circulated in Russia during 2020. Our analysis thus demonstrates that clade 2.3.4.4b H5N1 viruses have undergone complex reassortment with LPAI viruses in different geographic sites since 2020.

### Genotypic Analysis and Spread of Clade 2.3.4.4b H5N1 Viruses

Cui et al. analyzed 233 strains of clade 2.3.4.4b H5N1 viruses that were detected globally from October 1, 2020, to April 1, 2022, and revealed 16 genotypes (G01–16) (*17*). The 2 H5N1 viruses from Heilongjiang in this study belonged to G07, whereas the other 15 H5N1 viruses belonged to G10 (Appendix 2 Figure 1). Within 2 months of their detection in wild birds, viruses of these 2 genotypes spread to and were detected in domestic waterfowl in China (*17*).

Because the viruses are still spreading globally, we sought to gain further insight into the large-scale cross-continental transmission and reassortment patterns of clade 2.3.4.4b H5N1 viruses. To that end, we analyzed the sequences of 1,296 viruses that became available from April to October 2022 and detected an additional 19 genotypes (Appendix 1; Appendix 2 Table 3, Figure 4), including 5 in western Europe (G21, G28, G33, G34, and G35), 2 in eastern and central Europe (G19 and G20), 1 in Russia (G17), 1 in Africa (G18), and 10 in North America (G22, G23, G24, G25, G26, G27, G29, G30, G31, and G32) (Appendix 2 Table 3, Figure 5). We further grouped the available isolates into 11 distinct geographic regions, performed a phylogeographic analysis of their HA genes (Appendix 2 Table 3, Figure 5), and identified 22 spread pathways of clade 2.3.4.4b H5N1 viruses. Of note, 4 of those pathways were decisively supported with Bayes factors >1,000: 2 pathways showed G01 spreading from eastern and central Europe to Russia and from western Europe to North America; 1 showed G04 spreading from western Europe to southern Europe; and the fourth showed G07 spreading from eastern Asia to China (Appendix 2 Table 4). Those data indicate that Europe was the epidemic source for the global spread of clade 2.3.4.4b H5N1 viruses.

### Amino Acid Residues of Clade 2.3.4.4b H5N1 Viruses 

All 17 H5N1 viruses had residues 137A and 192I and did not have the glycosylation site at positions 158–160 in their HA (H3 numbering) (Appendix 2 Table 5), which have been reported to increase the affinity of avian influenza viruses for human-type receptors (*34*,*35*). The viruses also carried critical virulence-increasing residues, including 66S in PB1-F2 in 15 viruses; 30D, 43M, and 215A in M1 and 42S, 103F, and 106M in NS1 of all 17 viruses (*36*–*41*) (Appendix 2 Table 5). Therefore, clade 2.3.4.4b H5N1 viruses may have the capacity to infect and be virulent in humans.

### Virulence of H5N1 Wild Bird Viruses in Mice

In several countries, clade 2.3.4.4 H5 viruses have caused infections in humans and other mammals, including terrestrial small carnivores and marine mammals (Appendix 1). To evaluate the replication and virulence of wild bird H5N1 viruses in mammals, we tested 2 representative strains in BALB/c mice: MD/HLJ/HL-1/2021 in G07 and WS/HeN/14/2021 in G10. We found that both viruses replicated systemically and were detected in the nasal turbinates, lungs, spleens, and kidneys of the mice; MD/HLJ/HL-1/2021 was also detected in the brains of the mice (Figure 2, panel A). The LD_50_ value of MD/HLJ/HL-1/2021 in mice was 4.38 log_10_ EID_50_ and of WS/HeN/14/2021 was 5.17 log_10_ EID_50_ (Figure 2, panels B, C), indicating that H5N1 wild bird viruses are moderately pathogenic in mice (*1*,*5*,*7*,*9*).

### Lethality of H5N1 Wild Bird Viruses in Ducks

Many H5 viruses that are highly pathogenic to galliformes (e.g., chickens, quail, and turkeys) may still be of low pathogenicity to waterfowl (*1*,*15*). To investigate the replication and virulence of clade 2.3.4.4b H5N1 virus in waterfowl, we tested MD/HLJ/HL-1/2021 and WS/HeN/14/2021 in SPF ducks. We found that the 2 viruses replicated efficiently in ducks and were detected in all 9 investigated organs of ducks that were humanely killed on postinoculation day 3 (Figure 2, panel D). We detected virus shedding in oropharyngeal and cloacal swab samples from the infected ducks and contact ducks on postinoculation days 3 and 5 (Figure 2, panel E). MD/HLJ/HL-1/2021 led to the death of 4 of 5 inoculated and 3 contact ducks, whereas WS/HeN/14/2021 led to the death of all 5 inoculated and 3 contact ducks (Figure 2, panel F). Those data indicate that the 2 clade 2.3.4.4b H5N1 viruses we isolated from wild birds are lethal to ducks.

### Antigenic Matching of the H5 Vaccine Seed Virus Re14 with the H5N1 Wild Bird Viruses

To evaluate the antigenic difference between the emerged clade 2.3.4.4b of H5N1 viruses and the H5 vaccine strains, we generated antiserum in SPF chickens against 4 H5 vaccine strains (H5-Re11 [clade 2.3.4.4h], H5-Re12 [clade 2.3.2.1f], H5-Re13 [clade 2.3.4.4h], and H5-Re14 [clade 2.3.4.4b]) and tested their cross-reactive HI antibody titers to 4 representative viruses detected in this study: A/mandarin duck/Heilongjiang/HL-1/2021, A/whooper swan/Shanxi/608/2021, A/whooper swan/Henan/14/2021, and A/mandarin duck/Henan/426/2021 (Appendix 2 Table 7). We found that the 4 viruses reacted well with the antiserum of H5-Re14, with titers ranging from 64 to 128, less than 4-fold to the homologous titer, but reacted poorly with the antiserum of the other 3 vaccine strains (Figure 3, panel A; Appendix 2 Table 7).

We further evaluated the protective efficacy of the H5/H7 trivalent vaccine in chickens against challenge with a clade 2.3.4.4b H5N1 virus. Three weeks after vaccination, high titers of HI antibody to vaccine strain H5-Re14 and the H5N1 wild bird virus MD/HLJ/HL-1/2021 developed in the chickens (Figure 3, panel B). The vaccinated chickens were completely protected against MD/HLJ/HL-1/2021, with no virus shedding, and all chickens survived during the 14-day observation period (Figure 3, panels C, D); however, all control chickens died within 4 days of challenge, and the 7 chickens that were alive on postchallenge day 3 shed high titers of virus (Figure 3, panels C, D). Those data indicate that the vaccine currently used in China could provide solid protection against the clade 2.3.4.4b H5N1 viruses.

## Discussion

Clade 2.3.4.4b H5N8 virus caused massive outbreaks in poultry and wild birds in Europe, Africa, and Asia from January 2020 to October 2021 (*15*,*17*). During that time, the virus reassorted in countries or regions with different viruses and generated H5N1, H5N2, H5N3, H5N4, H5N5, and H5N6 viruses bearing the clade 2.3.4.4b HA gene (*4*,*14*,*16*,*17*,*23*). Among those new subtypes, only H5N1 viruses were widely spread by the movement of migratory birds and have caused thousands of outbreaks in Europe, Africa, Asia, and North America since their generation in the Netherlands in October 2020 (*4*,*14*,*17*). Our study revealed that the widely circulating H5N1 viruses have undergone complex reassortment with other LPAI viruses and formed >35 genotypes. Of those novel genotypes of H5N1 viruses, 10 were generated in North America since December 2021, when clade 2.3.4.4b H5N1 virus was first introduced to that region from western Europe (*18*). Given that the H5N1 reassortants found in Europe and North America have infected multiple wild mammals, their threat to public health should be carefully monitored and assessed.

Many countries in Europe and North America control HPAI by culling infected and suspected domestic birds, whereas China controls HPAI by using a massive vaccination strategy. The key to the success of the vaccination strategy is timely updates of vaccine strains (*15*,*32*,*33*). The currently used H5/H7 trivalent inactivated vaccine was updated and has been applied since January 2022; it is produced with 3 seed viruses: H5-Re13, targeting clade 2.3.4.4h H5 viruses; H5-Re14, targeting clade 2.3.4.4b H5 viruses; and H7-Re4, targeting H7N9 viruses (*33*). The antigenic assay in our study demonstrated that the H5-Re14 seed virus antigenically matches well with the recently emerged H5N1 wild bird viruses in China. The vaccine protective efficacy test also demonstrated that the novel H5/H7 trivalent vaccine provides solid protection against challenge with an emerging H5N1 isolate. The success of the mandatory vaccination policy for preventing and controlling HPAI virus in China is an example of a high-risk country using a vaccine to protect the poultry industry and of how vaccinating poultry can prevent and eliminate human infections with avian influenza virus, as evidenced by the successful control of H7N9 influenza (*32*,*33*,*42*). Therefore, we strongly recommend the use of vaccines to protect poultry from globally circulating 2.3.4.4b H5N1 viruses.

Appendix 1 Highly pathogenic avian influenza A (H5N1) virus information downloaded from GISAID (https://gisaid.org).

Appendix 2 Supplemental results from study of highly pathogenic avian influenza virus (H5N1) clade 2.3.4.4b introduced by wild birds, China, 2021.
