# Supplementary material for: Highly Pathogenic Avian Influenza Virus (H5N1) Clade 2.3.4.4b Introduced by Wild Birds, China, 2021
Source: Emerg Infect Dis. 2023 Jul;29(7):1367–75. doi: 10.3201/eid2907.221149 (PMC10310395; doi:10.3201/eid2907.221149)
Supplement: Appendix 2 — Supplemental results from study of highly pathogenic avian influenza virus (H5N1) clade 2.3.4.4b introduced by wild birds, China, 2021. [file 22-1149-Techapp-s2.pdf]

Article DOI: https://doi.org/10.3201/eid2907.221149

Highly Pathogenic Avian Influenza Virus (H5N1) Clade 2.3.4.4b Introduced by Wild Birds, China, 2021

Appendix 2

Appendix 2 Table 1. Clade 2.3.4.4b H5N1 HPAI viruses isolated from wild birds in China in 2021

Name	Abbreviation	Order	Time	Sample type (no.)	Total	Positive
A/mandarin duck/Heilongjiang/HL-1/2021	MD/HLJ/HL-1/2021	Anseriformes	Oct.2021	feces (324) swabs (113)	437	2
A/mandarin duck/Heilongjiang/HL-2/2021	MD/HLJ/HL-2/2021	Anseriformes				
A/whooper swan/Shanxi/14/2021	WS/SX/14/2021	Anseriformes	Dec.2021	feces (500)	500	4
A/whooper swan/Shanxi/20/2021	WS/SX/20/2021	Anseriformes				
A/whooper swan/Shanxi/601/2021	WS/SX/601/2021	Anseriformes				
A/whooper swan/Shanxi/608/2021	WS/SX/608/2021	Anseriformes				
A/whooper swan/Henan/2/2021	WS/HeN/2/2021	Anseriformes	Dec.2021	feces (800)	800	11
A/whooper swan/Henan/6/2021	WS/HeN/6/2021	Anseriformes				
A/whooper swan/Henan/8/2021	WS/HeN/8/2021	Anseriformes				
A/whooper swan/Henan/14/2021	WS/HeN/14/2021	Anseriformes				
A/whooper swan/Henan/15/2021	WS/HeN/15/2021	Anseriformes				
A/whooper swan/Henan/28/2021	WS/HeN/28/2021	Anseriformes				
A/whooper swan/Henan/44/2021	WS/HeN/44/2021	Anseriformes				
A/whooper swan/Henan/45/2021	WS/HeN/45/2021	Anseriformes				
A/whooper swan/Henan/46/2021	WS/HeN/46/2021	Anseriformes				
A/whooper swan/Henan/424/2021	WS/HeN/424/2021	Anseriformes				
A/mandarin duck/Henan/426/2021	MD/HeN/426/2021	Anseriformes				

Appendix 2 Table 2. The highest nucleotide homology of the whole genome for two H5N1 representative viruses.

Virus	Genotype	Segment	Phylogenic group	Closest viruses	Nucleotide identity (%)
MD/HL/HL-1/2021(H5N1)	G07	HA	Europe-H5N1 2020-2021	A/goose/Korea/H449/2020 (H5N8)	99.65
		NA	Europe-H5N1 2020-2021	A/turkey/Italy/21VIR8540-82/2021 (H5N1)	99.00
		PB2	Russia-LPAIV-2020	A/mallard/Novosibirsk region/3445k/2020 (H1N1)	99.30
		PB1	Russia-H5N8-2020	A/chicken/Czech Republic/1175-1/2020 (H5N8)	98.00
		PA	Europe-H5N1 2020-2021	A/Mallard/Netherlands/14/2021 (H5N1)	98.00
		NP	Europe-H5N1 2020-2021	A/turkey/England/057679/2021 (H5N1)	98.00
		M	Europe-H5N1 2020-2021	A/Bar-headed Goose/Tibet/XZ901/2021 (H5N1)	99.80
		NS	East Asia-LPAIV 2020	A/mallard/Novosibirsk region/3541k/2020 (H12N5)	99.40
WS/HeN/14 /2021(H5N1)	G10	HA	Europe-H5N1 2020-2021	A/eurasian teal/Netherlands/20016896-013/2020 (H5N1)	99.00
		NA	Europe-H5N1 2020-2021	A/duck/Bangladesh/19D1819/2021(H5N1)	99.00
		PB2	Russia-H5N8-2020	A/goose/Hunan/SE284/2022(H5N1)	99.00
		PB1	Europe-H5N1 2020-2021	A/chicken/Tyumen/27-40V/2021(H5N1)	99.00
		PA	Europe-H5N1 2020-2021	A/Cygnus columbianus/Hubei/121/2021(H5N1)	99.00
		NP	Europe-H5N1 2020-2021	A/duck/Bangladesh/19D1819/2021(H5N1)	99.00
		M	Europe-H5N1 2020-2021	A/turkey/Italy/21VIR8585-1/2021(H5N1)	99.00
		NS	Europe-H5N1 2020-2021	A/duck/Saratov/29-11V/2021(H5N1)	99.00

Appendix 2 Table 3. Geographic definitions for the regions used in this study

Continents	Regions	Countries	Virus (Total)	Genotype (virus number)
Europe	South Europe	Greece, Italy, Portugal, Spain	430	G01 (303), G04 (85), G05 (02), G08 (07), G12 (32), G11 (01)
	West Europe	Belgium, France, Germany, Ireland, Luxemburg, The Netherlands, United Kingdom	361	G01 (141), G02 (01), G04 (187), G08 (01), G11 (03), G15 (01), G16 (07), G21 (01), G28 (01), G33 (16), G34 (01), G35 (01)
	East and Central Europe	Albania, Belarus, Bulgaria, Croatia, Czech Republic, Hungary, Moldova, Poland, Romania, Russia (west of Ural Mountains), Slovenia	135	G01 (78), G04 (19), G05 (11), G08 (14), G11 (01), G12 (02), G13 (07), G19 (01), G20 (02)
	North Europe	Denmark, Estonia, Finland, Lithuania, Sweden	53	G01 (44), G04 (09)
Asia	East Asia	Japan, South Korea	23	G01 (20), G07(03),
	China		37	G01 (01), G07 (09), G09 (06), G10 (21)
	North Asia	Russia (east of Ural Mountains)	36	G01 (24), G03 (07), G05 (01), G06 (01), G10 (02), G17 (01),
	South Asia	Bangladesh	6	G14 (06)
	West Asia	Israel	2	G01 (02)
Africa	Africa	Niger, Benin, Senegal, Nigeria, Mali, Lesotho, Ghana, Burkina Faso	27	G01 (21), G18 (06)
North America	North America	USA, Canada	419	G01 (172), G22 (19), G23 (15), G24 (02), G25 (01), G26 (148), G27 (17), G29 (09), G30 (23), G31 (03), G32 (10)

Appendix 2 Table 4. Bayes factor and posterior probability for geographic spread

From	To	Bayes factor*	Posterior probability	Genotype
East and Central Europe	North Asia	6015.419813	0.994988167	G01
West Europe	South Europe	2062.449073	0.98552137	G04
East Asia	China	1783.415845	0.983293888	G07
West Europe	North America	1502.417593	0.980231101	G01
West Europe	East and Central Europe	359.7463069	0.922316581	G04
West Europe	North Europe	289.7673852	0.905332034	G04
East and Central Europe	South Europe	266.6247127	0.897953501	G01
South Europe	West Europe	202.2275488	0.869692329	G01
East and Central Europe	North Europe	158.1378828	0.839203675	G01
Africa	East and Central Europe	141.8880848	0.824028957	G01
West Europe	North Europe	116.4604235	0.793540303	G01
North Europe	East and Central Europe	69.99751646	0.697897814	G01
East and Central Europe	North Asia	67.8712242	0.691354587	G05
East and Central Europe	West Europe	58.39012651	0.658360017	G08
East and Central Europe	South Europe	57.35427561	0.654322706	G12
East and Central Europe	West Asia	43.9563981	0.591953223	G01
South Europe	East and Central Europe	41.98345781	0.580815815	G11
East and Central Europe	China	41.60138395	0.578588334	G01
South Europe	East and Central Europe	38.35787664	0.558680217	G08
East and Central Europe	East Asia	38.16350037	0.557427259	G01
West Europe	Africa	33.4508595	0.524711123	G01
North Asia	China	30.69927481	0.503271614	G10

Appendix 2 Table 5. Mutations detected in the clade 2.3.4.4b H5N1 viruses that contributed to increased binding to human-type receptors and virulence in mammals

		Amino acids in HA that may increase the affinity to human-type receptor (H3 numbering)				Mutations in different genes that may increase virulence in mice						
						PB1-F2	M1			NS1		
Virus	Genotype	S137A	N158D	T160A	T192I	N66S	N30D	I43M	T215A	P42S	L103F	I106M
MD/HLJ/HL-1/2021	G07	A	N	A	I	N	D	M	A	S	F	M
MD/HLJ/HL-2/2021	G07	A	N	A	I	N	D	M	A	S	F	M
WS/SX/14/2021	G10	A	D	A	I	S	D	M	A	S	F	M
WS/SX/20/2021	G10	A	D	A	I	S	D	M	A	S	F	M
WS/SX/601/2021	G10	A	D	A	I	S	D	M	A	S	F	M
WS/SX/608/2021	G10	A	D	A	I	S	D	M	A	S	F	M
WS/HeN/2/2021	G10	A	D	A	I	S	D	M	A	S	F	M
WS/HeN/6/2021	G10	A	D	A	I	S	D	M	A	S	F	M
WS/HeN/8/2021	G10	A	D	A	I	S	D	M	A	S	F	M
WS/HeN/14/2021	G10	A	D	A	I	S	D	M	A	S	F	M
WS/HeN/15/2021	G10	A	D	A	I	S	D	M	A	S	F	M
WS/HeN/28/2021	G10	A	D	A	I	S	D	M	A	S	F	M
WS/HeN/44/2021	G10	A	D	A	I	S	D	M	A	S	F	M
WS/HeN/45/2021	G10	A	D	A	I	S	D	M	A	S	F	M
WS/HeN/46/2021	G10	A	D	A	I	S	D	M	A	S	F	M
WS/HeN/424/2021	G10	A	D	A	I	S	D	M	A	S	F	M
MD/HeN/426/2021	G10	A	D	A	I	S	D	M	A	S	F	M

Appendix 2 Table 6. Clade 2.3.4.4b H5N1 outbreaks. Data downloads from WOAH (https://wahis.woah.org/#/dashboards/qd-dashboard)

Year	Semester	World region	Animal category	Outbreaks	Cases	Killed and disposed of	Deaths
2020	Jan-Jun	Africa	Wild	0	0	0	0
			Domestic	9	12,178	178,030	0
		Asia	Wild	11	217	0	217
			Domestic	16	20,597	25,075	18,074
	Jul-Dec	Africa	Wild	0	0	0	0
			Domestic	5	171,100	167,222	58,241
		Asia	Wild	0	0	0	0
			Domestic	11	2,957	9,930	1,896
		Europe	Wild	13	22	5	11
			Domestic	2	167	27,534	167
2021	Jan-Jun	Africa	Wild	56	1,585	578	1,482
			Domestic	63	762,858	2,740,292	355,189
		Asia	Wild	1	1	0	1
			Domestic	65	21,662	199,426	15,439
		Europe	Wild	82	262	1	261
			Domestic	4	30,507	56,209	9,326
	Jul-Dec	Africa	Wild	16	22,740	621	16,915
			Domestic	121	1,118,830	1,325,752	435,672
		Asia	Wild	39	8,513	1	8,438
			Domestic	65	142,464	3,144,530	80,090
		Europe	Wild	777	2,593	776	1,980
			Domestic	663	4,424,915	12,892,088	964,652
		Americas	Wild	11	18	8	8
			Domestic	2	344	109	328
2022	Jan-Jun	Africa	Wild	14	5,016	33	6,479
			Domestic	156	1,388,427	1,414,322	608,374
		Asia	Wild	143	560	0	547
			Domestic	248	206,996	5,606,772	242,085
		Europe	Wild	1,660	13,251	2,298	13,103
			Domestic	1,798	15,609,649	23,075,022	1,605,322
		Americas	Wild	225	1,038	230	1,144
			Domestic	487	94,597	42,181,259	274,446
	Jul-Dec	Africa	Wild	1	2	1	1
			Domestic	24	100,472	76,839	53,813
		Asia	Wild	4	275	0	275
			Domestic	81	86,838	1,280,501	72,685
		Europe	Wild	671	1,853	2,369	1,700
			Domestic	382	1,286,247	8,034,970	341,365
		Americas	Wild	43	40	8	35
			Domestic	285	288	8,790,840	277,716
Total				8,254	25,540,079	111,233,651	5,467,477

Appendix 2 Table 7. Cross-reactive hemagglutinin inhibition (HI) antibody titers of different H5N1 viruses with antisera of different vaccine strains

		Sera			
		Vaccine candidate			
		Clade 2.3.2.1f	Clade 2.3.4.4h	Clade 2.3.4.4b	
Clade	Antigen	Re12 CK/LN/SD007/2017 (H5N1)	Re13 DK/FJ/S1424/2020 (H5N6)	Re11 DK/GZ/S4184/2017 (H5N6)	Re14 WS/SX/4-1/2020 (H5N8)
2.3.2.1f	CK/LN/SD007/2017(H5N1) Re12	512	2	4	2
2.3.4.4h	DK/FJ/S1424/2020(H5N6) Re13	1*	1024	64	4
	DK/GZ/S4184/2017 (H5N6) Re11	1	128	256	8
2.3.4.4b	WS/SX/4-1/2020(H5N8) Re14	1	16	8	256
	MD/HLJ/HL-1/2021(H5N1)	1	8	16	64
	WS/HeN/14/2021(H5N1)	1	16	32	128
	WS/SX/608/2021(H5N1)	1	16	32	128
	MD/HeN/426/2021(H5N1)	2	16	32	128

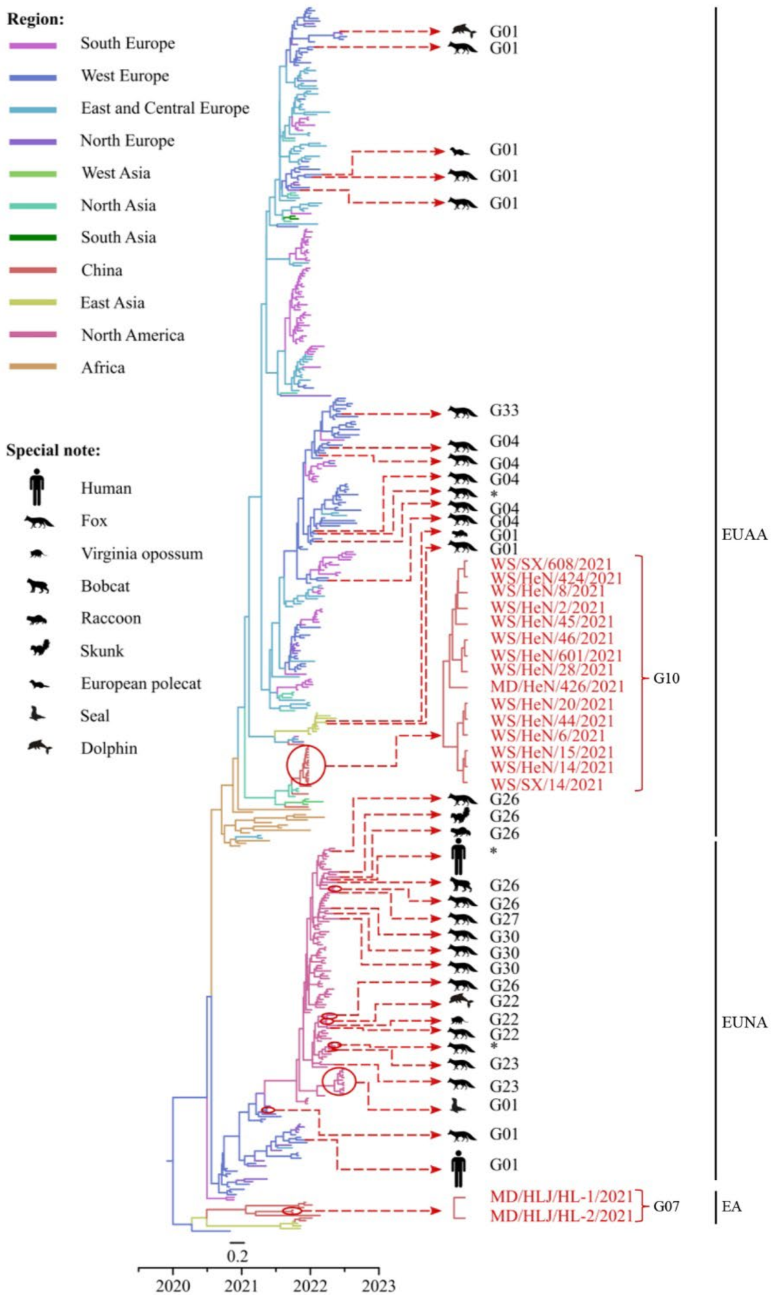

Appendix 2 Figure 1. Maximum-clade credibility time-scaled phylogenetic tree of the hemagglutinin genes of clade 2.3.4.4b H5N1 viruses. The trunk is colored according to geographic location. The detailed viral information used to generate the tree is shown in Appendix 1 (https://wwwnc.cdc.gov/EID/article/29/7/22-1149-App1.xlsx) and Appendix 2 Figure 2. The names of the 17 viruses isolated and sequenced in this study are shown in red. *, genotype is unknown because its complete genomic sequence is unavailable.

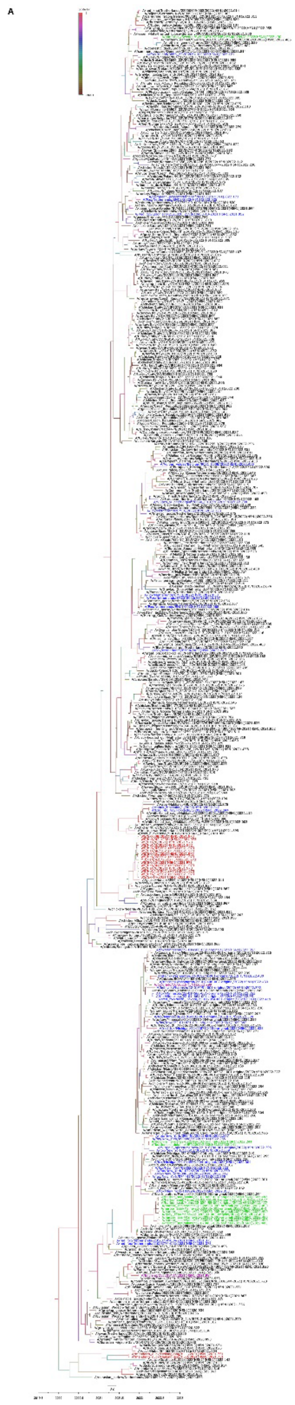

Appendix 2 Figure 2. The maximum-clade credibility tree of the HA gene was constructed by using the package BEAST (v1.10.2). A) Tree branch is colored for posterior probability; B) Tree branch is colored for genotype. The H5N1 viruses isolated in this study are colored in red. The H5N1 viruses isolated from humans, mammals, and marine mammals are shown in purple, blue, and green, respectively.

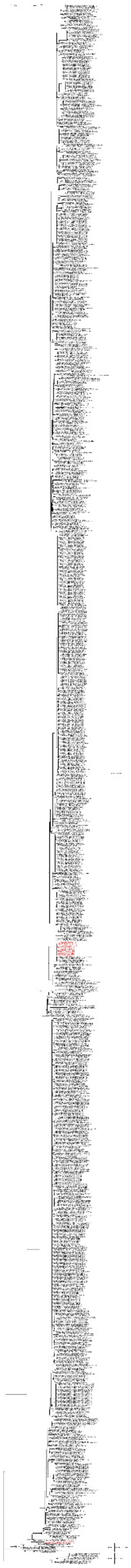

Appendix 2 Figure 3. Phylogenic analysis of the NA and internal genes of H5N1 viruses by using the neighbor-joining method. (A) NA, (B) PB2, (C) PB1, (D) PA, (E) NP, (F) M, (G) NS. The H5N1 viruses isolated in this study are shown in red.

Genotype	Reassortment	Region	PB2	PB1	PA	HA	NP	NA	M	NS
G01 (806)	AAAAAAAA	AF, WEU, NEU, ECEU,SEU, CN, EAS, EAS, WAS, NA	A-Group 1 (EU-H5N1 2021)	A-Group 1 (EU-H5N1 2021)	A-Group 1 (EU-H5N1 2021)	A-2.3.4.4b	A-Group 1 (EU-H5N1 2021)	A-Group 1 (EU-H5N1 2021)	A-Group 1 (EU-H5N1 2021)	A-Group 1 (EU-H5N1 2021)
G02 (1)	DBAADBAC	WEU	D-Group 4 (RU-H5N8 2020)	B-Group2 (EAS-LP 2015~2020)	A-Group 1 (EU-H5N1 2021)	A-2.3.4.4b	D-Group4 (EU-LP 2015~2021)	B-Group2 (EU-H1N1 2015~2020)	A-Group 1 (EU-H5N1 2021)	C-Group3 (RU-H5N8 2020)
G03 (7)	ABAAAAAA	NAS	A-Group 1 (EU-H5N1 2021)	B-Group2 (EAS-LP 2015~2020)	A-Group 1 (EU-H5N1 2021)	A-2.3.4.4b	A-Group 1 (EU-H5N1 2021)	A-Group 1 (EU-H5N1 2021)	A-Group 1 (EU-H5N1 2021)	A-Group 1 (EU-H5N1 2021)
G04 (300)	CAAAAAAA	WEU, NEU, ECEU, SEU	C-Group3 (RU-LP 2018~2019)	A-Group 1 (EU-H5N1 2021)	A-Group 1 (EU-H5N1 2021)	A-2.3.4.4b	A-Group 1 (EU-H5N1 2021)	A-Group 1 (EU-H5N1 2021)	A-Group 1 (EU-H5N1 2021)	A-Group 1 (EU-H5N1 2021)
G05 (14)	FAAAAAAA	ECEU, SEU, NAS	F-group6 (RU-LP 2019~2020)	A-Group 1 (EU-H5N1 2021)	A-Group 1 (EU-H5N1 2021)	A-2.3.4.4b	A-Group 1 (EU-H5N1 2021)	A-Group 1 (EU-H5N1 2021)	A-Group 1 (EU-H5N1 2021)	A-Group 1 (EU-H5N1 2021)
G06 (1)	DBDAAAAA	NAS	D-Group 4 (RU-H5N8 2020)	B-Group2 (EAS-LP 2015~2020)	D-Group 4 (RU-LP 2018~2019)	A-2.3.4.4b	A-Group 1 (EU-H5N1 2021)	A-Group 1 (EU-H5N1 2021)	A-Group 1 (EU-H5N1 2021)	A-Group 1 (EU-H5N1 2021)
G07 (12)	FCAAAAAB	EAS, CN	F-group6 (RU-LP 2019~2020)	C-Group3 (RU-H5N8 2020)	A-Group 1 (EU-H5N1 2021)	A-2.3.4.4b	A-Group 1 (EU-H5N1 2021)	A-Group 1 (EU-H5N1 2021)	A-Group 1 (EU-H5N1 2021)	B-Group2 (EAS-LP 2015~2020)
G08 (22)	BDAAAAAD	WEU, ECEU, SEU	B-Group 2 (EU-LP 2010~2015)	D-Group4 (EU-LP <2010)	A-Group 1 (EU-H5N1 2021)	A-2.3.4.4b	A-Group 1 (EU-H5N1 2021)	A-Group 1 (EU-H5N1 2021)	A-Group 1 (EU-H5N1 2021)	D-Group4 (EU-LP 2010~2015)
G09 (6)	DBBAACAC	CN	D-Group 4 (RU-H5N8 2020)	B-Group2 (EAS-LP 2015~2020)	B-Group2 (EAS-LP 2015~2020)	A-2.3.4.4b	A-Group 1 (EU-H5N1 2021)	C-Group3 (EAS-H1N1 2020)	A-Group 1 (EU-H5N1 2021)	C-Group3 (RU-H5N8 2020)
G10 (23)	DAAAAAAA	NAS, CN	D-Group 4 (RU-H5N8 2020)	A-Group 1 (EU-H5N1 2021)	A-Group 1 (EU-H5N1 2021)	A-2.3.4.4b	A-Group 1 (EU-H5N1 2021)	A-Group 1 (EU-H5N1 2021)	A-Group 1 (EU-H5N1 2021)	A-Group 1 (EU-H5N1 2021)
G11 (5)	BAAAAAAA	WEU, ECEU, SEU	B-Group 2 (EU-LP 2010~2015)	A-Group 1 (EU-H5N1 2021)	A-Group 1 (EU-H5N1 2021)	A-2.3.4.4b	A-Group 1 (EU-H5N1 2021)	A-Group 1 (EU-H5N1 2021)	A-Group 1 (EU-H5N1 2021)	A-Group 1 (EU-H5N1 2021)
G12 (34)	FBAAAAAA	ECEU, SEU	F-group6 (RU-LP 2019~2020)	B-Group2 (EAS-LP 2015~2020)	A-Group 1 (EU-H5N1 2021)	A-2.3.4.4b	A-Group 1 (EU-H5N1 2021)	A-Group 1 (EU-H5N1 2021)	A-Group 1 (EU-H5N1 2021)	A-Group 1 (EU-H5N1 2021)
G13 (7)	GACABAAD	ECEU	G-Group 7 (EAS-LP 2015~2020)	A-Group 1 (EU-H5N1 2021)	C-Group3 (EU-LP 2015~2020)	A-2.3.4.4b	B-Group2 (EAS-LP 2010~2015)	A-Group 1 (EU-H5N1 2021)	A-Group 1 (EU-H5N1 2021)	D-Group4 (EU-LP 2010~2015)
G14 (6)	EAAAAAAA	SAS	E-Group5 (EAS-LP 2010~2015)	A-Group 1 (EU-H5N1 2021)	A-Group 1 (EU-H5N1 2021)	A-2.3.4.4b	A-Group 1 (EU-H5N1 2021)	A-Group 1 (EU-H5N1 2021)	A-Group 1 (EU-H5N1 2021)	A-Group 1 (EU-H5N1 2021)
G15 (1)	BAAABAAA	WEU	B-Group 2 (EU-LP 2010~2015)	A-Group 1 (EU-H5N1 2021)	A-Group 1 (EU-H5N1 2021)	A-2.3.4.4b	B-Group2 (EAS-LP 2010~2015)	A-Group 1 (EU-H5N1 2021)	A-Group 1 (EU-H5N1 2021)	A-Group 1 (EU-H5N1 2021)
G16 (7)	AAAACAAA	WEU	A-Group 1 (EU-H5N1 2021)	A-Group 1 (EU-H5N1 2021)	A-Group 1 (EU-H5N1 2021)	A-2.3.4.4b	C-Group 3 (EU-LP 2010~2020)	A-Group 1 (EU-H5N1 2021)	A-Group 1 (EU-H5N1 2021)	A-Group 1 (EU-H5N1 2021)
G17 (1)	AAAABAAA	NAS	A-Group 1 (EU-H5N1 2021)	A-Group 1 (EU-H5N1 2021)	A-Group 1 (EU-H5N1 2021)	A-2.3.4.4b	B-Group2 (EAS-LP 2010~2015)	A-Group 1 (EU-H5N1 2021)	A-Group 1 (EU-H5N1 2021)	A-Group 1 (EU-H5N1 2021)
G18 (6)	AAGAAAAA	AF	A-Group 1 (EU-H5N1 2021)	A-Group 1 (EU-H5N1 2021)	G-Group7 (AF-H9N2 2016~2019)	A-2.3.4.4b	A-Group 1 (EU-H5N1 2021)	A-Group 1 (EU-H5N1 2021)	A-Group 1 (EU-H5N1 2021)	A-Group 1 (EU-H5N1 2021)
G19 (1)	FBAAAAAB	ECEU	F-group6 (RU-LP 2019~2020)	B-Group2 (EAS-LP 2015~2020)	A-Group 1 (EU-H5N1 2021)	A-2.3.4.4b	A-Group 1 (EU-H5N1 2021)	A-Group 1 (EU-H5N1 2021)	A-Group 1 (EU-H5N1 2021)	B-Group2 (EAS-LP 2015~2020)
G20 (2)	CACBAAAB	ECEU	C-Group3 (RU-LP 2018~2019)	A-Group 1 (EU-H5N1 2021)	C-Group3 (EU-LP 2015~2020)	A-2.3.4.4b	B-Group2 (EAS-LP 2010~2015)	A-Group 1 (EU-H5N1 2021)	A-Group 1 (EU-H5N1 2021)	B-Group2 (EAS-LP 2015~2020)
G21 (1)	ABAACAAA	WEU	A-Group 1 (EU-H5N1 2021)	B-Group2 (EAS-LP 2015~2020)	A-Group 1 (EU-H5N1 2021)	A-2.3.4.4b	C-Group 3 (EU-LP 2010~2020)	A-Group 1 (EU-H5N1 2021)	A-Group 1 (EU-H5N1 2021)	A-Group 1 (EU-H5N1 2021)
G22 (19)	HEHAEAAA	NA	H-Group8 (NA-LP 2015~2020 Group A)	E-Group5 (NA-LP 2017~2020)	H-Group8 (NA-LP 2018~2021)	A-2.3.4.4b	E-Group5 (NA-LP 2015~2021)	A-Group 1 (EU-H5N1 2021)	A-Group 1 (EU-H5N1 2021)	A-Group 1 (EU-H5N1 2021)
G23 (15)	HEAAEAAA	NA	H-Group8 (NA-LP 2015~2020 Group A)	E-Group5 (NA-LP 2017~2020)	A-Group 1 (EU-H5N1 2021)	A-2.3.4.4b	E-Group5 (NA-LP 2015~2021)	A-Group 1 (EU-H5N1 2021)	A-Group 1 (EU-H5N1 2021)	A-Group 1 (EU-H5N1 2021)
G24 (2)	HHAAEAAA	NA	H-Group8 (NA-LP 2015~2020 Group A)	H-Group8 (NA-unknown)	A-Group 1 (EU-H5N1 2021)	A-2.3.4.4b	E-Group5 (NA-LP 2015~2021)	A-Group 1 (EU-H5N1 2021)	A-Group 1 (EU-H5N1 2021)	A-Group 1 (EU-H5N1 2021)
G25 (1)	AHAAAAAA	NA	A-Group 1 (EU-H5N1 2021)	H-Group8 (NA-unknown)	A-Group 1 (EU-H5N1 2021)	A-2.3.4.4b	A-Group 1 (EU-H5N1 2021)	A-Group 1 (EU-H5N1 2021)	A-Group 1 (EU-H5N1 2021)	A-Group 1 (EU-H5N1 2021)
G26 (148)	HAAAEAAA	NA	H-Group8 (NA-LP 2015~2020 Group A)	A-Group 1 (EU-H5N1 2021)	A-Group 1 (EU-H5N1 2021)	A-2.3.4.4b	E-Group5 (NA-LP 2015~2021)	A-Group 1 (EU-H5N1 2021)	A-Group 1 (EU-H5N1 2021)	A-Group 1 (EU-H5N1 2021)
G27 (17)	IAAAEAAA	NA	I-Group9 (NA-LP 2015~2020 Group B)	A-Group 1 (EU-H5N1 2021)	A-Group 1 (EU-H5N1 2021)	A-2.3.4.4b	E-Group5 (NA-LP 2015~2021)	A-Group 1 (EU-H5N1 2021)	A-Group 1 (EU-H5N1 2021)	A-Group 1 (EU-H5N1 2021)
G28 (1)	CAEAAAAA	WEU	C-Group3 (RU-LP 2018~2019)	A-Group 1 (EU-H5N1 2021)	E-Group5 (EU-LP 2010~2015)	A-2.3.4.4b	A-Group 1 (EU-H5N1 2021)	A-Group 1 (EU-H5N1 2021)	A-Group 1 (EU-H5N1 2021)	A-Group 1 (EU-H5N1 2021)
G29 (9)	AAAAEAAA	NA	A-Group 1 (EU-H5N1 2021)	A-Group 1 (EU-H5N1 2021)	A-Group 1 (EU-H5N1 2021)	A-2.3.4.4b	E-Group5 (NA-LP 2015~2021)	A-Group 1 (EU-H5N1 2021)	A-Group 1 (EU-H5N1 2021)	A-Group 1 (EU-H5N1 2021)
G30 (23)	IFAAEAAE	NA	I-Group9 (NA-LP 2015~2020 Group B)	F-Group6 (NA-LP 2015~2016)	A-Group 1 (EU-H5N1 2021)	A-2.3.4.4b	E-Group5 (NA-LP 2015~2021)	A-Group 1 (EU-H5N1 2021)	A-Group 1 (EU-H5N1 2021)	E-Group5 (NA-LP 2015~2021)
G31 (3)	KGIAFAAE	NA	K-Group11 (NA-LP 2015~2020 Group D)	G-Group7 (NA-LP 2018)	I-Group9 (NA-LP 2015~2018)	A-2.3.4.4b	F-Group6 (NA-LP 2016~2018)	A-Group 1 (EU-H5N1 2021)	A-Group 1 (EU-H5N1 2021)	E-Group5 (NA-LP 2015~2021)
G32 (10)	JAAEAAA	NA	J-Group10 (NA-LP 2015~2020 Group C)	A-Group 1 (EU-H5N1 2021)	A-Group 1 (EU-H5N1 2021)	A-2.3.4.4b	E-Group5 (NA-LP 2015~2021)	A-Group 1 (EU-H5N1 2021)	A-Group 1 (EU-H5N1 2021)	A-Group 1 (EU-H5N1 2021)
G33 (16)	CAFAGAAF	WEU	C-Group3 (RU-LP 2018~2019)	A-Group 1 (EU-H5N1 2021)	F-Group6 (EU-H13 2012~2021)	A-2.3.4.4b	G-Group7 (EA-H13 2012~2021)	A-Group 1 (EU-H5N1 2021)	A-Group 1 (EU-H5N1 2021)	F-Group6 (EU-H13 2012~2021)
G34 (1)	AAFABAAB	WEU	A-Group 1 (EU-H5N1 2021)	A-Group 1 (EU-H5N1 2021)	F-Group6 (EU-H13 2012~2021)	A-2.3.4.4b	B-Group2 (EAS-LP 2010~2015)	A-Group 1 (EU-H5N1 2021)	A-Group 1 (EU-H5N1 2021)	B-Group2 (EAS-LP 2015~2020)
G35 (1)	CBCAAAAA	WEU	C-Group3 (RU-LP 2018~2019)	B-Group2 (EAS-LP 2015~2020)	C-Group3 (EU-LP 2015~2020)	A-2.3.4.4b	A-Group 1 (EU-H5N1 2021)	A-Group 1 (EU-H5N1 2021)	A-Group 1 (EU-H5N1 2021)	A-Group 1 (EU-H5N1 2021)

AF: Africa, WEU: West Europe, NEU: North Europe, ECEU: East and Central Europe,SEU: South Europe, CN: China, NAS: North Asia, EAS: East Asia, WAS: West Asia, SAS: South Asia, NA:North America, RU: Russia, EU: Europe, LP: low pathogenic avian influenza.

Appendix 2 Figure 4. The genotypes of the 1529 H5N1 viruses analyzed in this study. The groups for each segment are indicated by different colored bars.

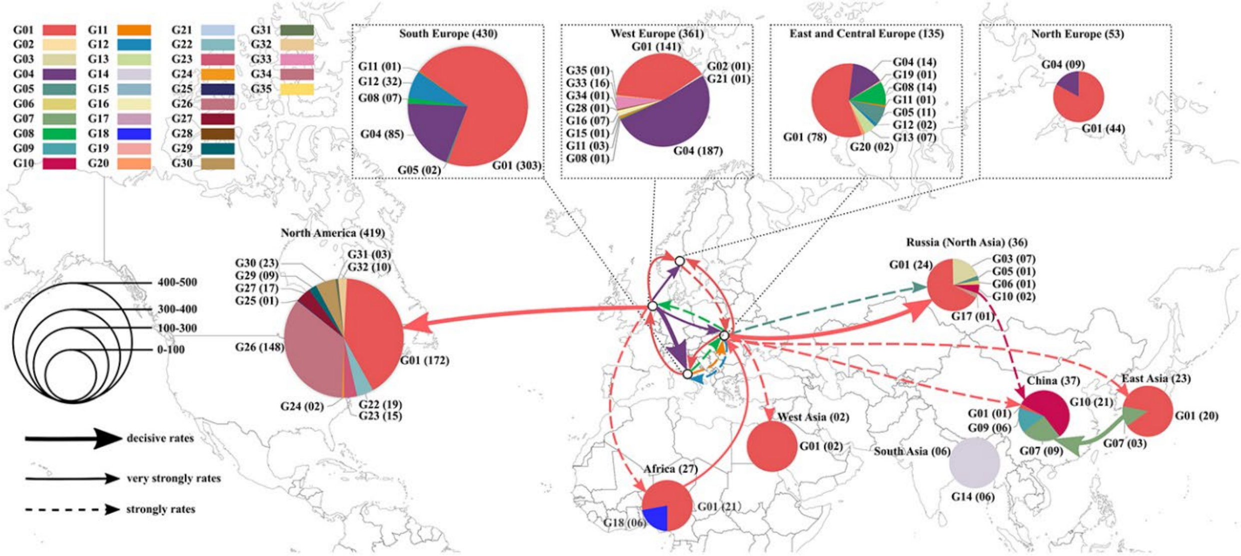

Appendix 2 Figure 5. Spatiotemporal spread and distribution of different genotypes of highly pathogenic avian influenza virus (H5N1) viruses bearing the clade 2.3.4.4b HA gene. The arrows in boldface indicate decisively supported diffusion (Bayes factor [BF] ≥1,000), solid arrows represent strongly supported diffusions (100 ≤BF <1,000), and dashed arrows show supported diffusions (30 ≤BF <100). The circle size is proportional to the number of isolates, and the numbers of strains of each genotype reported in the indicated region are shown in the parentheses.
